# T cell-mediated Immune response and correlates of inflammation and their relationship with COVID-19 clinical severity: not an intuitive guess

**DOI:** 10.1186/s12879-024-09490-y

**Published:** 2024-06-20

**Authors:** Nathalia Mantovani Pena, Luiz Claudio Santana, James R Hunter, Vinicius Fontanesi Blum, Tania Vergara, Celso Gouvea, Elcio Leal, Nancy Bellei, Mauro Schechter, Ricardo Sobhie Diaz

**Affiliations:** 1 Infectious Diseases Division, Federal University of São Paulo (UNIFESP), Pedro de Toledo, 669, Vila Clementino, Sao Paulo, SP, 04039-032 Brazil; 2 Oncohiv, Rio de Janeiro, Brazil; 3 Centro de Hematologia e Hemoterapia do Ceará, Fortaleza, CE, Brazil; 4 Laboratório de Diversidade Viral, Instituto de Ciências Biológicas, Universidade Federal do Pará, Belem, Pará, Brazil; 5 Universidade Federal do Rio de Janeiro (UFRJ), Rio de Janeiro, RJ, Brazil; 6 Weill Cornell Medicine, New York, United States of America

**Keywords:** COVID-19 severity, Cellular immune response, Antiviral response, HIV-1

## Abstract

**Background:**

Predictors of the outcome of Severe Acute Respiratory Syndrome Coronavirus 2 (SARS-CoV-2) infection remain to be fully determined. We evaluated selected viral characteristics and immunological responses that might predict and/or correlate to the clinical outcome of COVID-19.

**Methods:**

For individuals developing divergent clinical outcomes, the magnitude and breadth of T cell-mediated responses were measured within 36 h of symptom onset. Peripheral Blood Mononuclear Cells (PBMCs) were subjected to in vitro stimulation with SARS-CoV-2-based peptides. In addition, SARS-CoV-2 sequences were generated by metagenome, and HLA typing was performed using Luminex technology.

**Findings:**

CD4^+^ T cell activation was negatively correlated with SARS-CoV-2 basal viral load in patients with severe COVID-19 (*p* = 0·043). The overall cellular immune response, as inferred by the IFN-γ signal, was higher at baseline for patients who progressed to mild disease compared to patients who progressed to severe disease (*p* = 0·0044). Subjects with milder disease developed higher T cell responses for MHC class I and II-restricted peptides (*p* = 0·033).

**Interpretation:**

Mounting specific cellular immune responses in the first days after symptom onset, as inferred by IFN-γ magnitude in the ELISPOT assay, may efficiently favor a positive outcome. In contrast, progression to severe COVID-19 was accompanied by stronger cellular immune responses, higher CD4 + T cell activation, and a higher number of *in silico* predicted high-affinity class I HLA alleles.

**Supplementary Information:**

The online version contains supplementary material available at 10.1186/s12879-024-09490-y.

## Introduction

The heterogeneity of the COVID-19 disease course remains one of the most intriguing aspects of infection by SARS-CoV-2, its etiological agent. Soon after the beginning of the pandemic, the Chinese Center for Disease Control and Prevention reported that approximately 80% out of 44,672 COVID-19 cases consisted of mild disease [1]. Disease severity was associated with advanced age, male gender, socioeconomic factors, and underlying medical conditions such as hypertension, diabetes, cardiovascular, and chronic pulmonary diseases [2–5].

It is known that SARS-CoV-2 infection induces the production of a cascade of cytokines that promotes platelet activation and a series of clotting factors and thrombus, as seen in pulmonary arterioles in autopsies of fatal cases [6]. In addition, it has been suggested that dysregulation in the acute response that stems from an impaired type I interferon response may lead to severe disease [7–10].

There are data to suggest a link between disease severity and disturbed T and B cell-mediated immune responses [11, 12]. HLA variability may also be associated with clinical outcomes since, for instance, an association between HLA haplotype and risk of respiratory insufficiency has been reported [13]. This might be due to the ability to present conserved epitopes of SARS-CoV-2 [14] or due to the presence of ancestral protective HLA alleles that may contribute to resistance to severe forms of the disease [15].

In the present paper, we investigated the association of selected viral characteristics with markers of immune response early in the course of the infection and how this pattern correlates to progression to severe forms of the disease. The natural history of HIV infection and its relationship with viral load and immune response early in the infection is well documented [16, 17]. We thus used stored samples from a group of treatment-naïve people living with HIV (PLHIV) to compare and contrast with the relationship between viral load, immune response, and disease progression in early SARS-CoV-2 infection.

## Methods

The biological samples used in the study were obtained from three different convenience populations. For the first group, we used biological samples from 50 participants in a clinical trial who were recruited in São Paulo, Brazil between May 20th and September 21st, 2020 [18]. (ClinicalTrials.gov NCT04348409) (Supplementary Table 1 A). In brief, 50 hospitalized patients (18 years or older) in non-critical condition, with PCR-confirmed COVID-19, mild respiratory insufficiency (Saturation O_2_ inferior to 95%), and a maximum of 36 h since symptom-onset were included in that trial. The 36-hour post-symptom onset threshold was arbitrarily chosen to maximize the probability to observe any potential antiviral effect of the drug that was being tested since SARS-CoV-2 plasma viral load is expected to decay thereafter as the disease evolves progressively. The inclusion of participants occurred during the first wave of COVID-19, before vaccination started. According to their clinical progression, participants were subsequently classified as severe (requiring admission to an intensive care unit for mechanical ventilation and/or death during hospitalization, 19 patients) or non-severe cases (31 patients). Samples from baseline and follow-up period (21 days after initial diagnosis) were used to investigate correlates of inflammation (T cell activation markers) and SARS-COV-2 viral loads, cell mediate immune responses, and disease progression.

The second group consisted of 20 patients with PCR-confirmed COVID-19, diagnosed during March and April 2020 (the patients characteristics are presented in Table 1). Samples were collected at the time of diagnosis. Patients were prospectively classified as having mild, moderate, or severe/critical disease according to the following criteria. Mild disease was defined as patients who were never admitted to the hospital. Moderate disease was defined as patients who were admitted to the hospital within ten days of diagnosis with evidence of lower respiratory tract disease, with an oxygen saturation measured by pulse oximetry ≥ 95% on room air, but who did not progress to severe disease. Severe disease, in turn, was defined as patients who were admitted or transferred to the ICU for invasive oxygen therapy and/or who died within ten days of diagnosis due to respiratory failure, shock, or multiorgan dysfunction. Samples collected from these patients were used to investigate the possible role of HLA alleles and the SARS-CoV-2 genetic profile (metagenomics) in disease progression.

**Table 1:** Characteristics of randomly selected patients with COVID-19 according to the disease severity (group 2), SARS-CoV-2 viral load, age, HLA profile, and metagenome sequencing data revealing the SARS-CoV-2 lineage. Ct: Cycle threshold; F: Female; M: Male; bp: Base pairs

Sample	Ct	Severity	Age	Gender	Sequence (bp)	Reads	Depth	Pango Lineage
1	16	Mild	29	F	29.901	3,031,594	14,129x	B.1.1.28
2	16	Mild	58	F	29.902	6,074,044	28,277x	B.1.1.28
3	16	Mild	53	M	29.903	2,038,126	9,415x	B.1.1.28
4	17	Mild	32	F	29.903	2,312,867	10,777x	B.1.1.33
5	18	Mild	72	M	29.866	106,300	495x	B.1.1.33
6	18	Mild	24	F	29.901	1,693,627	7,858x	B.1.1.33
7	18	Mild	NA	M	29.9	631,093	2,927x	B.1.1.33
8	18	Mild	27	M	29.836	28,576	134x	B.1.1.28
9	14	Moderate	71	M	29.896	4,162,231	19,705x	B.1.1.28
10	16	Moderate	63	M	29.882	204,526	965x	B.1.1.33
11	17	Moderate	46	M	29.866	207,577	982x	B.1.1.28
12	17	Moderate	61	F	29.903	5,488,065	24,854x	B.1.1.28
13	18	Moderate	68	F	29.663	7,627	38x	B.1.1.28
14	20	Moderate	67	F	29.874	31,205	143x	B.1.1.28
15	20	Moderate	27	F	29.848	18,282	86x	B.1.1.28
16	23	Moderate	38	M	29.867	430,916	2,039x	B.1.1.28
17	14	Severe	75	M	29.902	992,684	4,654x	B.1.1.28
18	16	Severe	63	F	29.896	1,440,742	6,848x	B.1.1.28
19	18	Severe	81	M	29.865	148,897	697x	B.1.1.33
20	19	Severe	44	F	29.517	7,701	38x	B.1.1 0.28

	HLA I			HLA II				
Sample	HLA-A	HLA-B	HLA-C	DRB1	DQA1	DQB1	DPA1	DPB1
1	02:01/02:01	44:03/50:01	06:02/16:01	04:05/07:01	02:01/03:01	02:02/03:02	01:03/02:01	04:01/11:01
2	29:02/74:01	07:02/15:10	07:01/08:04	09:01/13:01	01:03/03:01	02:02/06:03	01:03/01:03	02:01/03:01
3	01:01/23:01	44:02/44:03	04:01/05:01	04:02/07:01	02:01/03:01	02:02/03:02	01:03/02:02	01:01/04:01
4	01:02/23:01	42:01/44:03	04:01/17:01	07:01/11:01	01:02/02:01	02:02/06:02	02:01/02:02	01:01/14:01
5	24:02/25:01	15:01/18:01	03:03/07:01	11:04/15:01	01:02/05:05	03:01/06:02	01:03/02:01	04:01/17:01
6	02:01/23:01	51:01/53:01	01:02/06:02	13:01/13:02	01:02/03:01	03:03/06:04	01:03/01:03	03:01/04:02
7	02:01/24:02	07:02/15:04	03:03/07:02	03:01/15:01	01:02/05:01	02:01/06:02	01:03/01:03	02:01/04:01
8	01:01/29:02	08:01/44:03	07:01/16:01	03:01/07:01	02:01/05:01	02:01/02:02	01:03/01:03	02:01/03:01
9	02:01/02:01	15:11/15:18	03:03/08:01	04:05/09:01	03:02/03:03	03:03/04:01	02:01/02:02	02:01/03:01
10	02:01/24:02	07:02/44:03	04:01/07:02	04:01/07:01	02:01/03:03	02:02/03:02	01:03/01:03	04:01/04:01
11	24:02/32:01	15:01/58:01	03:04/07:18	01:01/03:01	01:01/05:05	03:01/05:01	02:01/02:01	01:01/10:01
12	02:01/23:01	44:02/50:02	04:01/05:01	04:05/14:01	01:01/03:01	03:02/05:03	01:03/01:03	03:01/04:01
13	02:01/23:17	44:03/58:01	14:02/15:05	04:05/15:03	01:02/03:03	02:02/06:02	01:03/03:01	03:01/105:01
14	30:02/74:01	14:02/57:03	07:01/08:02	13:03/15:03	01:02/02:01	02:02/06:02	01:03/04:01	02:01/105:01
15	30:01/31:01	35:02/39:09	04:01/07:02	08:02/11:04	04:01/05:05	03:01/04:02	01:03/01:03	03:01/04:02
16	01:01/23:01	07:02/35:02	04:01/07:02	11:02/11:04	01:03/05:05	03:01/06:03	01:03/02:01	01:01/03:01
17	24:02/68:01	35:03/35:03	04:01/04:01	04:01/07:01	02:01/03:03	02:02/03:02	02:01/02:01	11:01:/14:01
18	02:02/11:01	15:16/27:05	01:02/14:02	01:01/01:02	01:01/01:01	05:01/05:01	01:03/01:03	04:01/104:01
19	02:02/30:02	18:01/53:01	04:01/05:01	03:01/11:01	01:02/05:01	02:01/06:02	01:03/02:01	02:02/131:01
20	32:01/68:01	35:01/35:01	03:03/04:01	07:01/11:03	02:01/05:05	02:02/03:01	01:03/02:01	02:01/17:01

The third population were 30 treatment naïve (Supplementary table 1B), PLHIV who were enrolled to participate in a clinical trial of antiretroviral drugs we had conducted several years before and whose total blood, plasma, and peripheral blood mononuclear cells (PBMC) we had in storage. These samples were used to correlate CD4 + T cell activation, as inferred by the percentage of CD38 and HLA-DR in CD4 + T cells with HIV-1 viral load and CD4 + T cell counts [19].

SARS-CoV-2 infection was diagnosed by Polymerase Chain Reaction (PCR) of nasopharyngeal swabs collected no later than 36 h after symptom onset, according to patient’s declaration. The SARS-CoV-2 RT-PCR qualitative analyses were performed using the AllplexTM 2019-nCoV Assay, version 2.1, October 30th 30, 2020, according to the manufacturer’s instructions. SARS-CoV-2 RT-PCR viral load was performed as previously described [18], including the Ribosomal A Protein amplification as an internal human RNA control. Collected specimens were centrifuged at 5000 g for 5 min to remove debris. For RNA libraries, 240 µL of the supernatant was filtered through a 0·22 µM filter (Sigma Aldrich, Pittsburg, USA) for removal of host cells and bacteria, followed by RNA extraction using Linear Acrylamide (Thermo Fisher Scientific, San Jose, USA) and the QIAamp RNA Viral Mini Kit (Qiagen, Hilden, Germany), according to the manufacturer’s instructions. Nucleic acids were treated with DNase I and concentrated using the RNA Clean & Concentrator™-5 (ZymoResearch, Irvine, USA). Human and bacterial rRNA were depleted using the QIAseq Fast Select –rRNA HMR Kit (Qiagen, Hilden, Germany) and the QIAseq Fast Select − 5 S/16S/23S Kit (Qiagen, Hilden, Germany), respectively, according to the manufacturer’s instructions. Subsequently, RNA was reverse transcribed with 40 µmolar of primer A (5′-GTTTCCCACTGGAGGATANNNNNNNNN-3′) [20] and SuperScript III Reverse Transcriptase (ThermoFisher Scientific, San Jose, USA) followed by second-strand synthesis with Sequenase DNA polymerase (Thermo FisherSicentific, San Jose, USA). The cDNA was enriched by PCR with 100 µmolar of primer B (5′-GTTTCCCACTGGAGGATA-3′) [20] and Q5® High-Fidelity DNA Polymerase (New England Biolabs, Beverly, USA). Reactions were incubated as follows: 98 °C for 30 s, 25 cycles of 98 °C for 10 s, 54 °C for 30 s, and 72 °C for 30 s, followed by a final incubation of 72 °C for 2 min. PCR products were purified with Agencourt AMPure XP (BeckmanCoulter, Brea, USA) and 100 ng were subjected to RNA library preparation using QIAseq FX DNA Library Kit (Qiagen, Hilden, Germany), according to the manufacturer’s instructions. Sequencing was carried out through the high output 300 cycles run of the NextSeq 550 system (Illumina, San Diego, USA). The DNA sequences were submitted to the GeneBank and accession number are (pending). Raw data is available on request.

The HLA typing was performed for both class I (HLA-A, HLA-B, and HLA-C) and class II molecules (DRB1, DQA1, DQB1, DPA1, DPB1) through sequence-specific oligonucleotide probes (SSO) using LABType™ XR and CWD DNA Typing Test (One Lambda, Inc) in conjunction with the LABScan3D™ instrument (Luminex FLEXMAP 3D instrument).

Cell-mediated immunity was evaluated through the secretion of IFN-γ, in which cryopreserved PBMCs) were subjected to an enzyme-linked immunospot (ELISpot) assay (BD™ ELISPOT Human IFN-γ ELISPOT Kit). PBMCs were isolated on the same day that the oropharyngeal specimens were collected for diagnosis (baseline) and on the 10th and/or 21st day after diagnosis (unless the participant died before the date scheduled for collection) and immediately frozen. To conduct the Elispot assay, a Multiscreen® IP plate (Merck) was sensitized with 100 µL (10 µg/ mL) of anti-IFN- γ antibody in PBS. The plate containing the antibody was incubated at 4 °C for 24 h. After the sensitization step, the plate was washed once with RPMI culture medium containing 10% fetal bovine serum (FBS) and 2% penicillin/streptomycin. Finally, blocking was performed with 200 µL of culture medium (RPMI + 10% FBS and 2% antibiotic) per well and the plate was incubated for 2 h at room temperature. On the day the ELISpot was performed, cells were thawed and stimulated in vitro with peptides derived from the SARS-CoV-2 Wuhan wild-type sequence covering the Membrane (M), Nucleoprotein (S), and Spike (S) proteins. To conduct the Elispot, 300,000 cells were incubated with 0·5 μm of PepTivator® SARS-CoV-2 Prot_M, Prot_N, Prot_S, and select peptides (Miltenyi Biotec B.V. & Co. KG) consisting of 63 peptides MHC class I-restricted and 25 peptides class II-restricted originated from structural proteins (S, M, N, and E) and non-structural proteins of SARS-CoV-2. The PBMCs and peptides were incubated in a RPMI medium (Gibco™) containing 10% FBS and 1% Penicillin/Streptomycin at 37 °C for 24 h. 100 µL of the enzyme Streptavidin-Horseradish Peroxidase (BD™ ELISPOT Streptavidin-HRP) was pipetted into each well and the plate was incubated at room temperature for 1 h. 100 µL of substrate-containing solution was pipetted into each well. Color development was monitored for up to 60 min. The wells were read using an AID ELISpot Reader, version 7.0. All experiments were carried out in duplicate and the mean spot forming cells (SFC) from negative controls were subtracted from the mean SFC of the peptide stimulated wells. The ELISpot response was then calculated per million cells. The peptides used to stimulate the cells were selected based on their immunogenicity [21, 22]. Flow cytometry data from the cohort was obtained as previously reported [18]. Peripheral blood mononuclear cells (PBMC) were separated and cryopreserved. After thawing, PBMCs were labeled with anti-CD3 APC and anti-CD4 PercP for the lymphocytic subpopulation and anti-CD38 FITC and anti-HLA-DR. PE for cellular activation (BD Biosciences, San Diego CA, USA). Fixed cells were acquired on a FACSCalibur apparatus and analyzed with CellQuest software (BD Biosciences, San Diego CA, USA), and the analysis was performed with FlowJo v X 0.7. Results were expressed as mean fluorescent intensity (MFI). The gating strategy is depicted in Supplementary Fig. 1.

### Bioinformatics and statistical analysis

The raw reads were filtered based on quality (Phred score) and quality-trimmed using Trimmomatic (v. 0.38.1) [23], utilizing the following parameters: (1) trimming low-quality base (quality score < 20); (2) removing reads shorter than 70 bp; (3) tracing and cutting off sequencing adapters. Next, the human reads were filtered by mapping the reads against human genome reference GRChg38 using bowtie2 with default parameters. Then, short reads were *de novo* assembled into contigs using Megahit [24] and Trinity [25]. Finally, each contig was compared against the non-redundant nucleotide and protein Reference Viral Database (RVDB) (v22.0) [26] using blastx and blastn, respectively, to identify potential viruses.

To generate the full-length SARS-CoV-2 genome from each sample, the host-filtered fastq files were mapped against the Wuhan-Hu-1 reference genome (NC_045512.2) using Bowtie2 [27], and a consensus genome sequence was obtained from the aligned BAM file using iVar software [28]. To generate a consensus sequence, the iVar parameter was set with a minimum of ten reads supporting each genomic position. Coverage statistics were generated from the sorted BAM file using qualimap (v2.2.2-dev) [29]. Additionally, the epidemiological lineage classification of SARS-CoV-2 genomes was determined using the Pangolin COVID-19 Lineage Assigner Web server [30], which is available online at https://cov-lineages.org/index.html. The SARS-CoV-2 consensus sequence and HLA profile of each study participant served as input for the online tool Custommune [31], a pipeline designed to perform an *in silico* prediction of peptides able to induce both neutralizing antibodies and cell-mediated response against SARS-CoV-2 conserved epitopes.

R language (version 3.5.3) [32] was utilized for statistical analysis. Statistical significance of IFN-γ signal between groups of participants who progressed to severe or non-severe disease was calculated using the Wilcoxon rank-sum test. The Kruskal-Wallis rank-sum test and Wilcoxon rank-sum test with Benjamini-Hochberg correction for multiple comparisons were employed for comparing the IFN-γ signal among the peptides stimulations. Spearman correlation was performed using R scripts. The HLA frequencies and number of high-affinity alleles were calculated using GraphPad Prism version 9.0.0.

## Results

### Higher levels of inflammation, as inferred by the CD4 ^+^ T cell activation, are associated with lowering SARS-CoV-2 viral loads earlier in the disease among individuals that progressed to severe COVID-19

We analyzed samples from fifty hospitalized patients who participated in a clinical trial [18]. The inclusion criteria for this clinical trial were RT-PCR-confirmed COVID-19, mild respiratory insufficiency (Saturation O_2_ inferior to 95%), and a maximum of 36 h since symptom onset. Eight patients progressed to severe forms of COVID-19 and required intensive care and invasive oxygen therapy within days of admission; three of them died within ten days after diagnosis. At the end of follow-up on day 21 of the study, two of the five living patients who had progressed to severe forms of COVI-9 had a RT-PCR positive for SARS-Cov-2 compared to two out of 27 who had progressed to mild disease (Fisher exact test = NS).

**Fig. 1:**
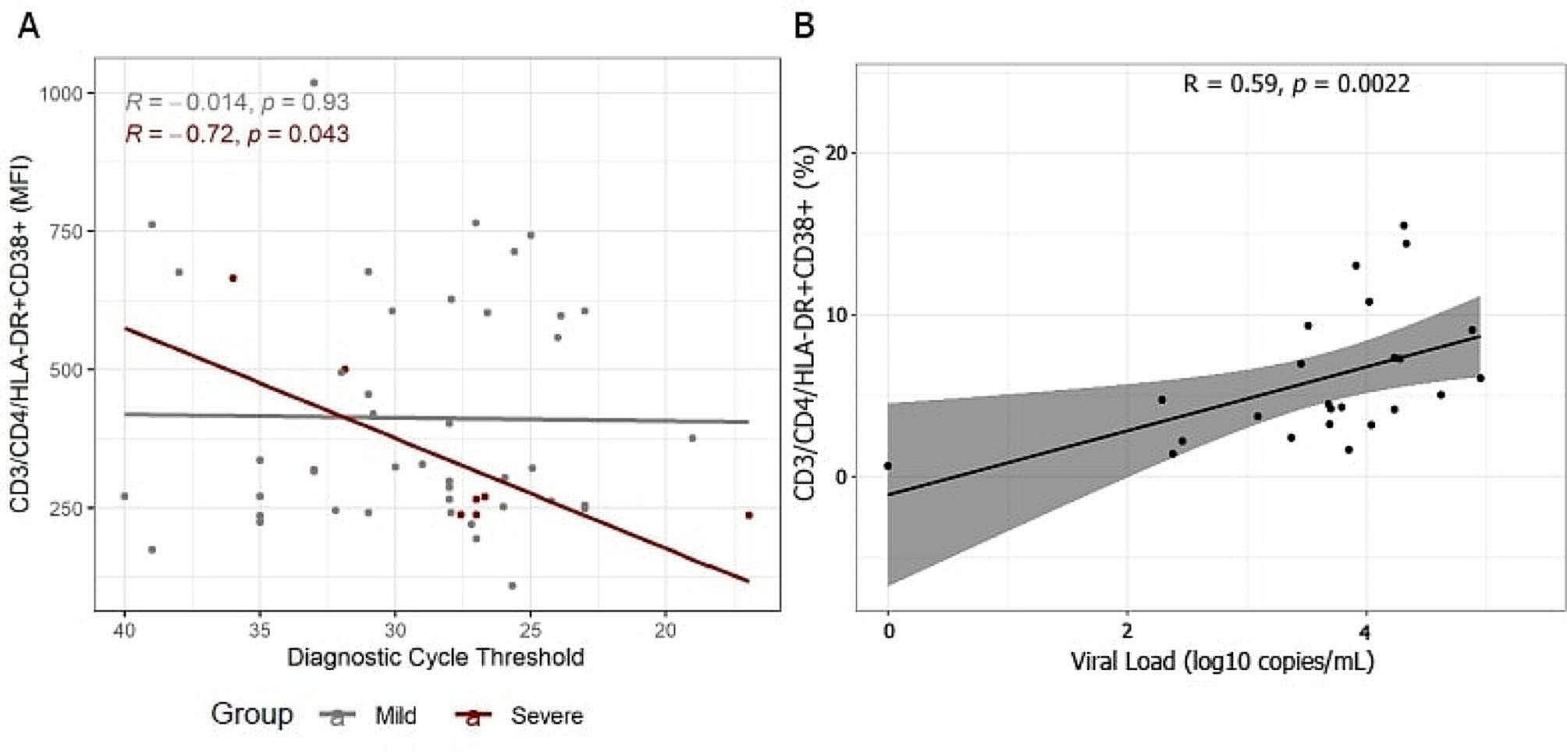
Correlation between T-cell activation markers HLA-DR and CD38 within 36 h of symptom onset Figure 1A: Spearman correlation between Ct values and T-cell activation markers in patients who progressed and who did not progress to severe forms of COVID-19 (group 1). Note that the scale of the horizontal axis is reversed to indicate greater viral load moving to the right. Figure 1B: Correlation between percentage of CD3^+^/CD4^+^DR + CD38 + T cells and HIV viral load in antiretroviral naïve PLHIV (group 3). MFI = Mean Fluorescence Intensity

Based on Mean Fluorescence Intensity (MFI), there was a negative correlation between viral load, measured by Ct values, and the T-cell activation markers HLA-DR and CD38 in CD4 + T cells among patients who progressed to severe disease at diagnosis (*R* = -0·72, *p* = 0·043; Fig. 1A). In other words, there was an inverse correlation between viral load at diagnosis and CD4^+^ T cell activation in subjects who progressed to severe disease. In contrast, there was no significant correlation between Ct values and CD4 + activation in individuals who did not progress to severe disease (*R* = -0·014, *p* = 0·93; Fig. 1A). We also analyzed samples from 30 treatment-naïve PLHIV [19]. In this case, a positive correlation was found between HIV viral load and T-cell activation, based on the percentage of CD3+/CD4 + DR + CD38 + T cells present (*R* = 0.59, *p* = 0.002; Fig. 1B).

### Cellular immune response as inferred by Elispot results, and progression to severe COVID-19

**Fig. 2:**
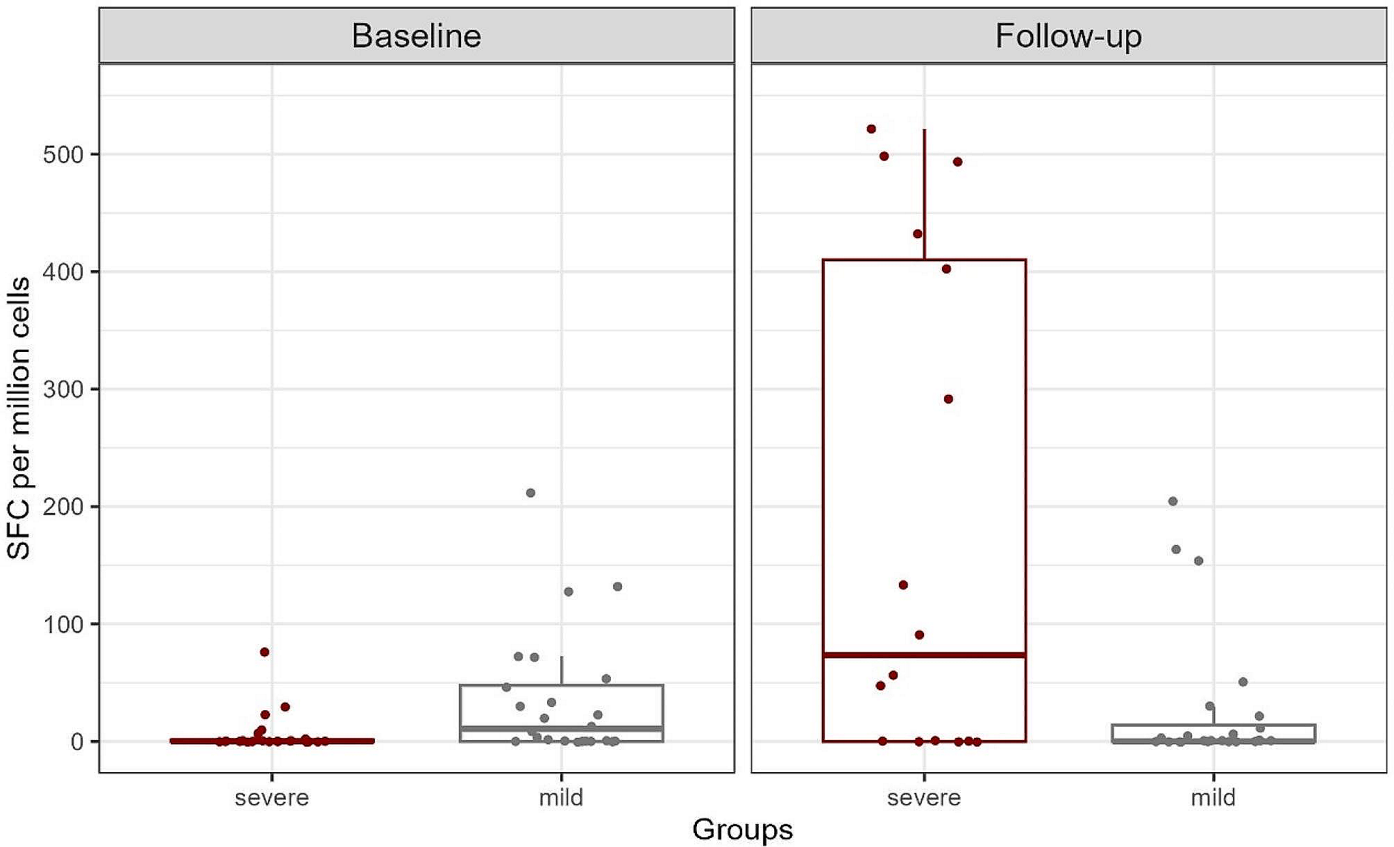
Comparison of total cell-mediated immune response between individuals who progressed and who did not progress to severe disease (group 1). Upon stimulation of cells with SARS-CoV-2 peptides, IFN-γ signals generated were calculated and compared. SFC: Spot forming cells

The overall Elispot analysis showed that individuals who progressed to severe COVID-19 induced significantly lower spot-forming cells at baseline than those who did not progress (medians, 0·00 and 10·72, 95% CI, 0·0000432 to 29·69, respectively, *p* = 0·0044). In contrast, the IFN-γ secretion was higher for those subjects developing a severe clinical outcome at follow-up (medians, 73·42 and 0·00, 95% CI, -292·05 to -0·0000222, respectively, *p* = 0·032) (Fig. 2). Therefore, individuals who progressed from moderate to severe COVID-19 had lower cellular immune response earlier in the disease and higher cellular immune response later in the disease than individuals who evolved from moderate to mild symptoms.

### Cellular immune response to SARS-CoV-2 specific peptides

**Fig. 3:**
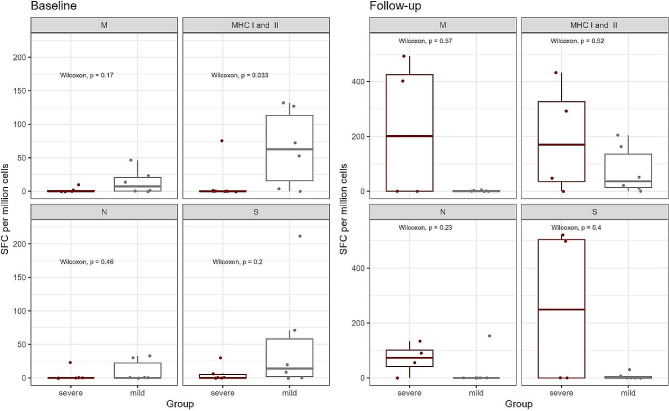
Antiviral immune response at baseline and follow-up (group 1). Once the cells were stimulated with SARS-CoV-2 peptides, the IFN-γ signals generated from each stimulation from samples from patients with mild and severe COVID-19 were compared. M: Membrane; MHC: Major Histocompatibility Complex; N: Nucleocapsid; S: Spike; SFC: Spot Forming Cells

**Fig. 4:**
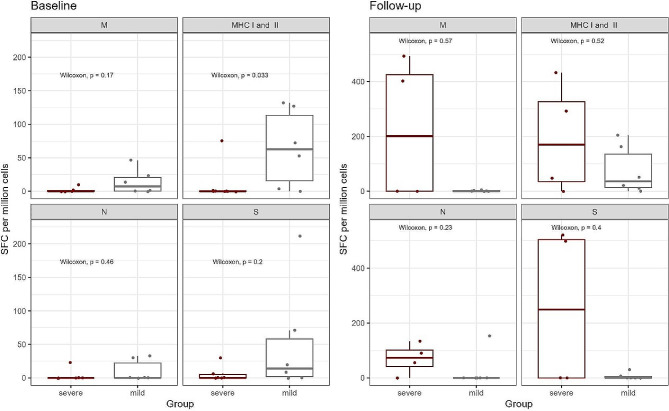
Antiviral immune response for progressors and non-progressors to severe forms of the disease at baseline and follow-up (group 1). Upon stimulation with SARS-CoV-2 peptides, the IFN-γ signals generated from each stimulation of samples from patients with mild and severe COVID-19 were compared concerning the baseline and follow-up time points. M: Membrane; MHC: Major Histocompatibility Complex; N: Nucleocapsid; S: Spike; SFC: Spot Forming Cells

To further understand if the stimulus induced by specific SARS-CoV-2 peptides reached different levels between groups at baseline, the IFN-γ responses for a distinct set of peptides were compared. Signals at baseline were higher in the group that evolved to mild COVID-19 only for cells stimulated with MHC class I and II-restricted peptides (*p* = 0·033) (Fig. 3). Conversely, the group that evolved to severe COVID-19 showed a trend towards an increase in the IFN-γ response for cells stimulated with MHC class I and II-restricted and N peptides (Fig. 4). Therefore, the increased cellular immune response associated with the evolution from moderate to mild COVID-19 relates to the MHC-restricted peptides response earlier in the disease.

### The higher the cellular SARS-CoV-2 immunity, the higher the inflammation

**Fig. 5:**
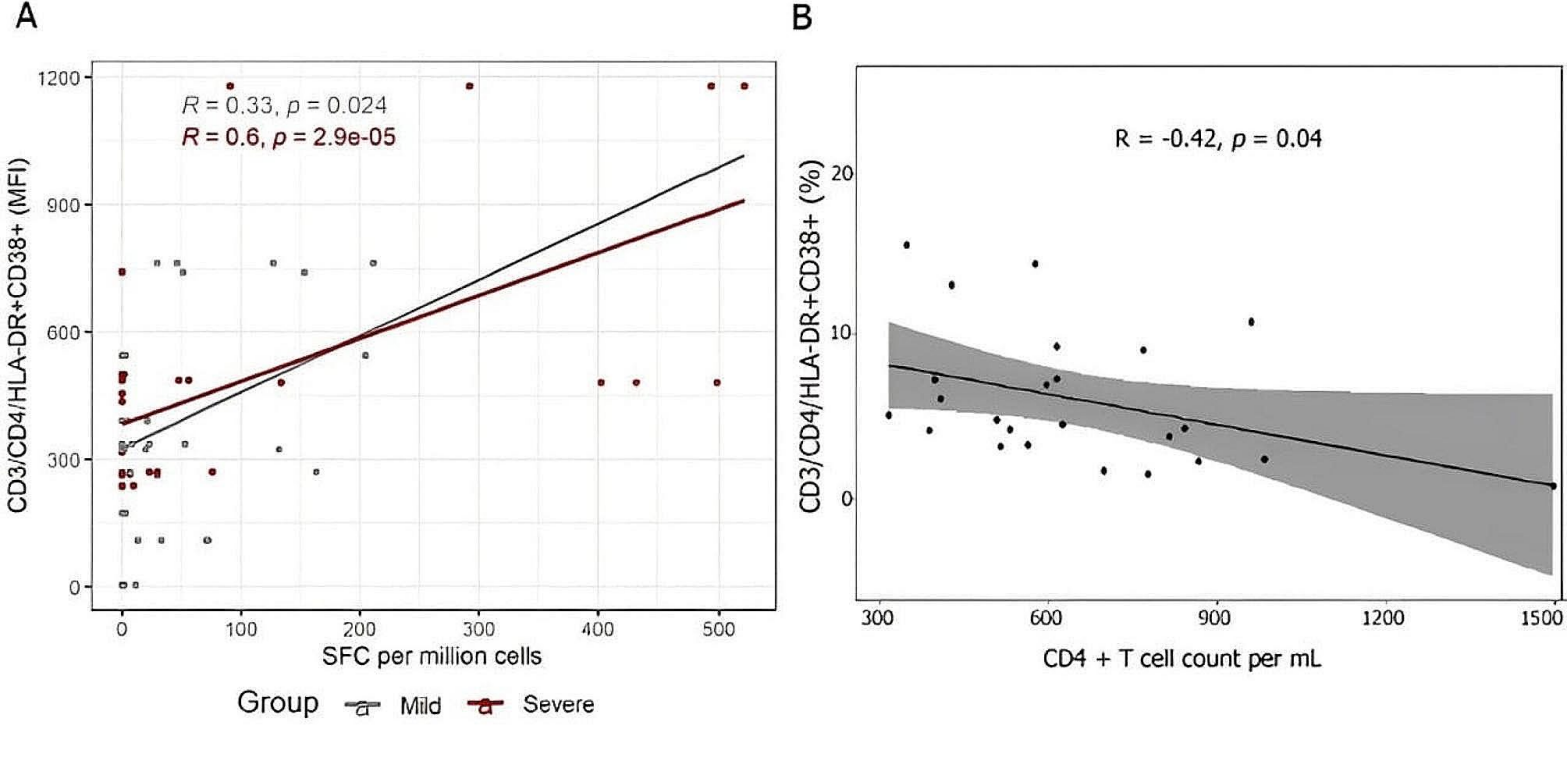
Association between cellular immune response and T cell activation. Figure 5A: The mean IFN-γ signal calculated for each peptide stimulation and the mean CD4^+^ T cell activation measured by the surface markers HLA-DR plus CD38 using Spearman correlation (group 1). Figure 5B: Spearman correlation of the mean CD4^+^ T cell activation and the mean CD4^+^ T cell counts among antiretroviral naïve PLHIV (group 3). MFI: Mean Fluorescence Intensity

Unlike baseline higher viral loads, which were negatively correlated with CD4^+^ T lymphocytes activation only in patients who progressed to severe disease, the IFN-γ signal induced by stimulated cells was positively associated with the CD4^+^ T activation for participants who did (*R* = 0·6, *p* < 0·01) or did not progress to severe disease (*R* = 0·33, *p* = 0·024; Fig. 5A). Again, to draw a parallel between immune response and magnitude of inflammation in HIV infection, we correlated cellular activation of CD4^+^ T cells and degree of immunodeficiency based on CD4^+^ T cell count in untreated PLHIV. Once more, the greater the HIV-induced immunodeficiency, the higher the cellular activation (*R* =-0·42, *p* = 0·04, Fig. 5B). In contrast to what is found in HIV, we found that the higher the cellular immunity against SARS-CoV-2, the higher the inflammation, as inferred by the CD4 + T cell activation.

### Higher predicted SARS-CoV-2 immune response among individuals with severe COVID-19

Finally, we sought to identify specific HLA alleles related to distinct clinical phenotypes at COVID-19 diagnosis in patients from another cohort (Table 1). Oropharyngeal samples used were from randomly selected patients. A group of 8 patients who presented for diagnosis and were classified as having mild or moderate COVID-19, and four patients with severe COVID-19 disease, according to the contemporary WHO classification, were included. The number of Human Leukocyte Antigen (HLA) alleles class I and II is shown in Supplementary Fig. 2.

**Fig. 6:**
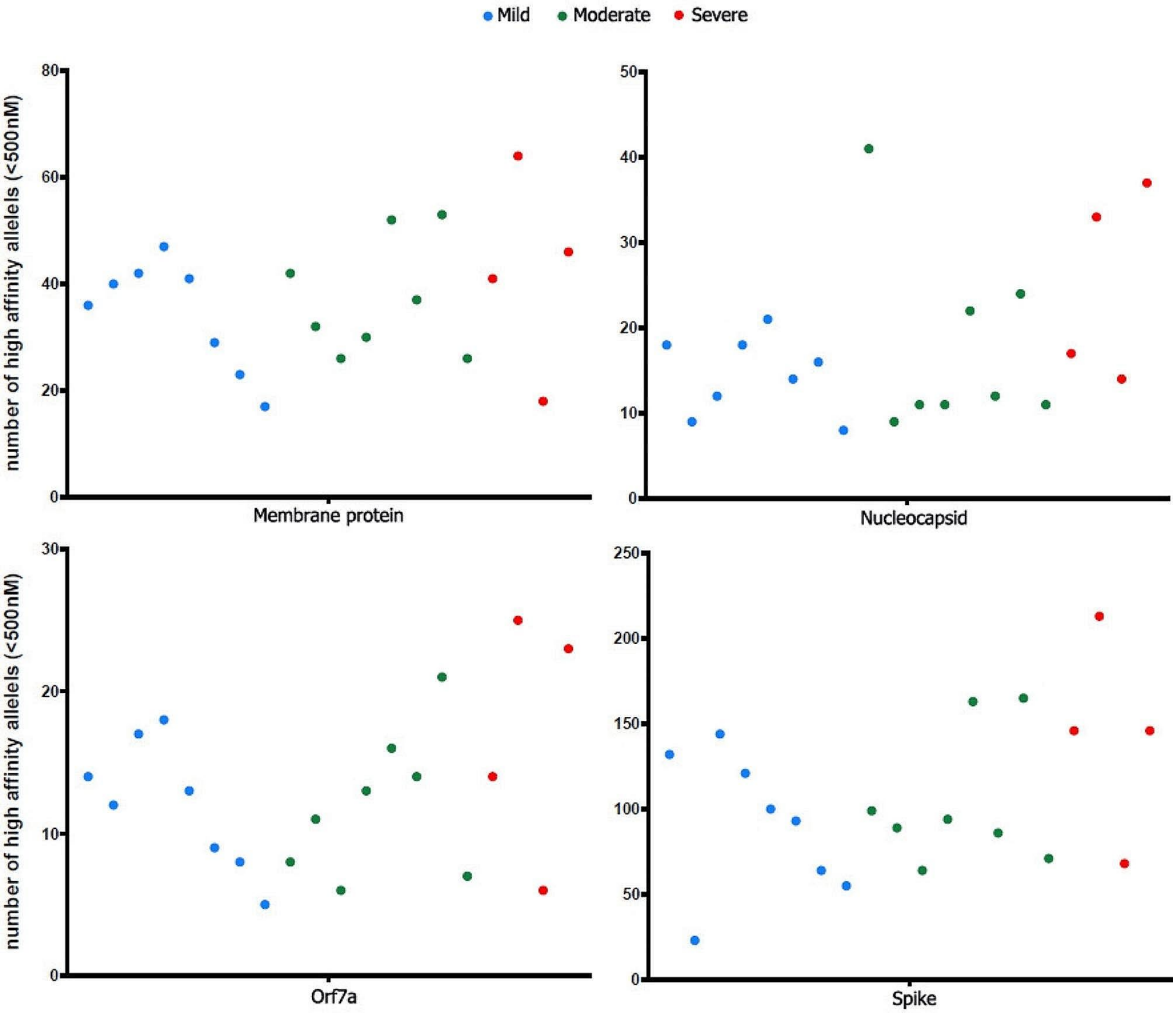
Number of high-affinity alleles for HLA class I molecules for Membrane, Nucleocapsid, Orf7a, and Spike proteins (group 2). Each dot represents the result of a specific individual depicted by distinct colors for mild, moderate and severe COVID-19. The HLA profile and SARS-CoV-2 consensus sequences were subjected to the Custommune pipeline [31]. Epitopes were predicted to have high binding affinity if the predicted IC50 < 500nM

Although some HLA alleles were present only in the groups of patients with mild and moderate disease, we could not discern a specific HLA or SARS-CoV-2 genomic sequence profile associated with disease severity. Upon submitting the SARS-CoV-2 consensus sequences and HLA alleles to the Custommune pipeline [31], it generated a set of peptides for HLA class I and II molecules predicted to bind conserved SARS-CoV-2 epitopes and their predicted affinity. The data showed a trend for a more significant number of high-affinity class I HLA alleles for patients with severe COVID-19 (Fig. 6). Therefore, higher immune response to SARS-CoV-2, as inferred by the predicted higher number of high-affinity class I HLA alleles to bind conserved SARS-CoV-2 epitopes, is present among individuals with severe COVID-19.

## Discussion

Predictors of SARS-CoV-2 infection outcome remain to be fully defined, particularly biomarkers. Moreover, genetic and immunological factors that trigger the so-called cytokine storm, a major determinant of COVID-19 outcome, are yet to be thoroughly characterized. Therefore, in the present paper, we sought to investigate selected immunological parameters early in the course of the disease that might be associated with progression to severe forms of COVID-19, defined as the need for invasive oxygen therapy and/or death in the first two weeks after symptom onset.

By and large, signs and symptoms of infectious diseases correlate with the ability of the host to produce inflammation [33]. Inflammation is usually beneficial to the host since it plays an important role in tissue repair and pathogen elimination. In general, severely immunocompromised individuals with limited ability to produce inflammation will suffer less tissue and organ damage despite an abundance of pathogens. A case in point is the HIV immune reconstitution syndrome (IRIS). In IRIS, once viral replication is suppressed by antiretroviral therapy, immunity rapidly recovers and the patient may experience extensive organ and tissue damage due to inflammation [34]. It should also be pointed out that the micro-inflammation that occurs in some chronic infections, such as HIV infection, may be associated with tissue and organ damage and with an acceleration of the aging process [35]. Accordingly, T-cell activation is a hallmark of HIV pathogenesis. Thus, immunophenotypic and serum markers have been used to quantify T-cell activation in PLHIV [36]. CD38 and HLA-DR, both in CD4^+^ and CD8^+^ T cells, are the best markers of inflammation in these individuals and correlated with ongoing viral replication, microbial translocation from the gastrointestinal tract, and the presence of co-infections [37, 38].

Like HIV infection, data indicate that higher SARS-CoV-2 viral loads at diagnosis are associated with poorer COVID-19 prognosis [39, 40]. In the present study, we found a positive correlation between viral load at diagnosis and prognosis but an inverse correlation between viral load and immune activation and inflammation at diagnosis. On the other hand, this situation was reversed within three weeks after symptom onset, i.e., a positive correlation was found between immune activation and inflammation and progression to severe disease.

In the early stages of SARS-CoV-2 infection, a robust T-helper response is beneficial. T-helper cells play a crucial role in coordinating the immune response by activating other immune cells and producing cytokines. A strong T-helper response helps control viral replication and spread by activating cytotoxic T cells and facilitating the production of antibodies. Additionally, an effective T-helper response might help mitigate disease progression by promoting the clearance of infected cells and reducing the severity of symptoms.

As the infection progresses, especially in cases where the virus persists or the immune response is dysregulated, an overly strong T-helper response may occur. This could lead to a cytokine storm, where pro-inflammatory cytokines are excessively released, leading to tissue damage and more severe disease outcomes. The association between stronger T cell immunity and higher T cell activation in individuals progressing to severe disease strengthens the notion that the magnitude of the humoral response correlates with the outcome [41, 42], given that orchestration by T helper cells is crucial for developing neutralizing antibodies [43]. We hypothesize that in some cases, the hyperactive immune response may become maladaptive, causing harm to the host rather than effectively combating the virus. The balance between an effective antiviral immune response and the risk of immunopathology, with immune-mediated tissue damage, is delicate and can shift over the course of infection. Therefore, understanding the timing and dynamics of immune responses to SARS-CoV-2 is critical for developing targeted therapeutic interventions and vaccines that modulate the immune system appropriately to prevent severe disease outcomes while maintaining protective immunity.

In summary, while a robust T-helper response initially controls viral replication and disease progression in COVID-19, an overly strong and dysregulated immune response later in the course of infection might contribute to more severe outcomes. This underscores the complexity of the immune response to SARS-CoV-2 and highlights the importance of timing in understanding and managing COVID-19 immunopathology.

HLA haplotypes have also been associated with COVID-19 prognosis [13, 15, 44]. Our observations suggest that mounting HLA type 1 and 2 robust immune responses but not to nucleocapsid and spike proteins early in the course of the infection is important for controlling it. On the other hand, a robust immune response later in the course of the infection appears to be associated with more severe disease. This is further reinforced by the *in-silico* analysis utilizing the HLA profile of patients matched to the SARS-CoV-2 genome of their infecting viruses, in which patients who evolved to less severe disease have a stronger ability to recognize and present viral epitopes that are more likely to activate a T cell response.

The small sample size and the lack of information about underlying medical conditions in the second group of patients are some of the limitations of the present study. Nonetheless, our findings indicate an association between immune response very early in the course of the disease and outcome. This association contrasts with what is seen in HIV infection since low SARS-CoV-2 viral loads up to 36 h from symptom onset were associated with high T-cell activation and better outcome.

Our findings appear to be counter-intuitive and contradict published data that indicated that the presence of HLA class I molecules with higher binding affinity is associated with a more favorable COVID-19 outcome [45]. Nonetheless, data from clinical trials seem to lend support to our findings since the use of corticosteroids, which have immune modulatory and anti-inflammatory effects, early in the course of the disease does not confer clinical benefit and may even be harmful, whereas their use later in the course of the disease in patients with poorer prognosis is associated with a better outcome [46, 47]. We believe that our observations may help identify biomarkers for predicting disease progression and assist in the development of immunomodulatory therapeutic interventions and the appropriate timing of their use.

### Electronic supplementary material

Below is the link to the electronic supplementary material.

Supplementary Material 1

Supplementary Material 2

Supplementary Material 3

## Data Availability

All data for this study has been included in manuscript figures, tables, and online supplemental information. The DNA sequences were submitted to the GeneBank and accession number are (pending). Raw data is available on request (please contact James R Hunter at jameshunterbr@gmail.com).
