# Supplementary material for: T cell-mediated Immune response and correlates of inflammation and their relationship with COVID-19 clinical severity: not an intuitive guess
Source: BMC Infect Dis. 2024 Jun 20;24:612. doi: 10.1186/s12879-024-09490-y (PMC11191252; doi:10.1186/s12879-024-09490-y)
Supplement: Supplementary file 1 — Supplementary Material 1 [file 12879_2024_9490_MOESM1_ESM.docx]

Supplementary Table 1A

| **Sample ID** | **Age** | **VL** | **CD4** | **CD8** | **CD4/CD8** | **CD4/CD38+HLA-DR+** | **CD8/CD38+HLA-DR+** |
| --- | --- | --- | --- | --- | --- | --- | --- |
| **1** | 45 | 76739 | 768,00 | 2138,00 | 0,36 | 9.07 | 26.18 |
| **3** | 22 | 76505 | 511,00 | 1048,00 | 0,49 | 5.45 | 11.85 |
| **5** | 48 | 15789 | 610,00 | 1038,00 | 0,59 | 9.07 | 17.16 |
| **7** | 35 | 32428 | 564,00 | 1415,00 | 0,40 | 8.14 | 31.28 |
| **9** | 35 | 102440 | 585,00 | 2219,00 | 0,26 | 33.02 | 50.37 |
| **11** | 58 | 42652 | 316,00 | 843,00 | 0,37 | 5.06 | 34.92 |
| **13** | 44 | <50 | 1496,00 | 2367,00 | 0,63 | 0.67 | 3.29 |
| **15** | 45 | 239 | 777,00 | 872,00 | 0,89 | 1.41 | 6.05 |
| **21** | 31 | 813 | 508,00 | 853,00 | 0,60 | 4.75 | 13.99 |
| **23** | 33 | 10566 | 961,00 | 849,00 | 1,13 | 10.83 | 39.46 |
| **25** | 35 | 8066 | 427,00 | 684,00 | 0,62 | 13.06 | 24.21 |
| **27** | 26 | 4898 | 563,00 | 757,00 | 0,74 | 4.68 | 8.8 |
| **29** | 33 | 20572 | 348,00 | 1017,00 | 0,34 | 14.16 | 43.96 |
| **31** | 27 | 18895 | 398,00 | 1298,00 | 0,31 | 6.23 | 18.1 |
| **33** | 29 | 10906 | 514 | 902,00 | 0,57 | 12.48 | 18.54 |
| **37** | 44 | 289 | 867 | 780,00 | 1,11 | 2.2 | 7.38 |
| **2** | 41 | 2370 | 984 | 1194 | 0,82 | 2.41 | 10.79 |
| **6** | 34 | 249346 | 392 | 1010 | 0,39 | 6.53 | 16,00 |
| **8** | 29 | 35909 | 488 | 1434 | 0,34 | 3.21 | 12.03 |
| **12** | 38 | 89588 | 407 | 1114 | 0,37 | 6.09 | 40.74 |
| **14** | 36 | 2882 | 597 | 883 | 0,68 | 6.98 | 25.08 |
| **16** | 27 | 4794 | 625 | 1196 | 0,52 | 4.49 | 17.32 |
| **18** | 36 | 21798 | 576 | 693 | 0,83 | 14.42 | 31.89 |
| **20** | 21 | 3245 | 615 | 288 | 2,14 | 9.34 | 21.02 |
| **22** | 34 | 17119 | 388 | 893 | 0,43 | 4.16 | 21.75 |
| **24** | 30 | 5033 | 531 | 511 | 1,04 | 5.27 | 13.72 |
| **28** | 34 | 7030 | 699 | 1321 | 0,53 | 2.19 | 16.13 |
| **32** | 28 | 12579 | 615 | 1314 | 0,47 | 7.49 | 15.69 |
| **34** | 24 | 6279 | 843 | 1575 | 0,54 | 6.1 | 14.44 |
| **36** | 27 | 1233 | 815 | 925 | 0,88 | 2.86 | 12.24 |

Supplementary Table 1B

Supplementary Table 1: ***Characteristics of selected patients from groups 1 and 3*.** Table 1A: Baseline characteristics of the first group of patients hospitalized with moderate COVID-19 during the first wave [18]. CT: Cycle threshold of RT-PCR for SARS-COV-2; F: Female; M: Male; ICU: Admitted to an Intensive Care Unit; *non-invasive ventilation. CD38 and HLA-DR in CD4 and CD8 T cells are presented in percentages. Table 1B: Characteristics of the third group analyzed, consisting of antiretroviral naïve male people living with HIV [19]. VL: HIV RNA viral load; CD4: CD4+ T cell counts; CD38 and HLA-DR in CD4 and CD8 T cells are presented in percentages.
