# Supplementary figures and images for: T cell-mediated Immune response and correlates of inflammation and their relationship with COVID-19 clinical severity: not an intuitive guess
Source: BMC Infect Dis. 2024 Jun 20;24:612. doi: 10.1186/s12879-024-09490-y (PMC11191252; doi:10.1186/s12879-024-09490-y)

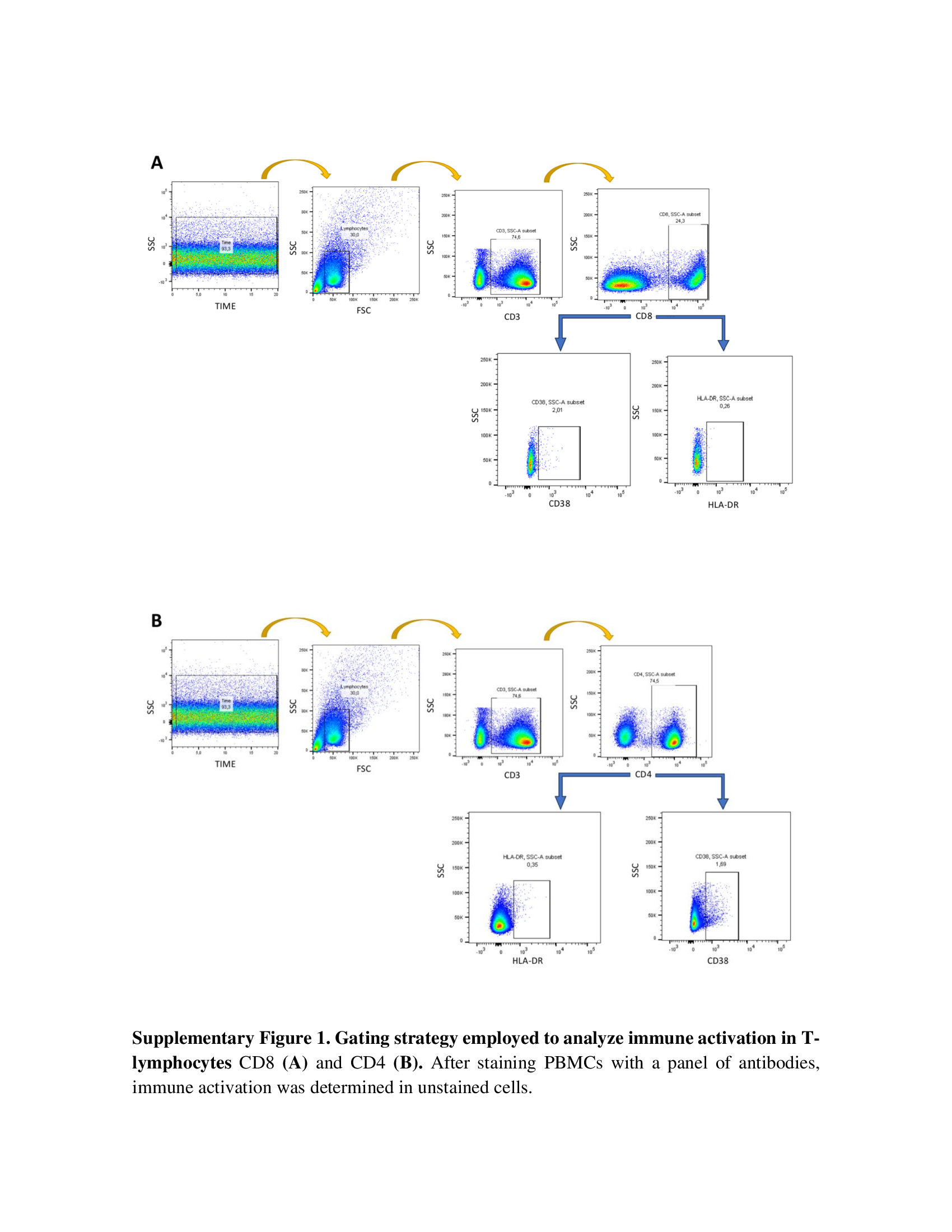

Supplement: Supplementary file 2 — Supplementary Material 2 [file 12879_2024_9490_MOESM2_ESM.jpg]

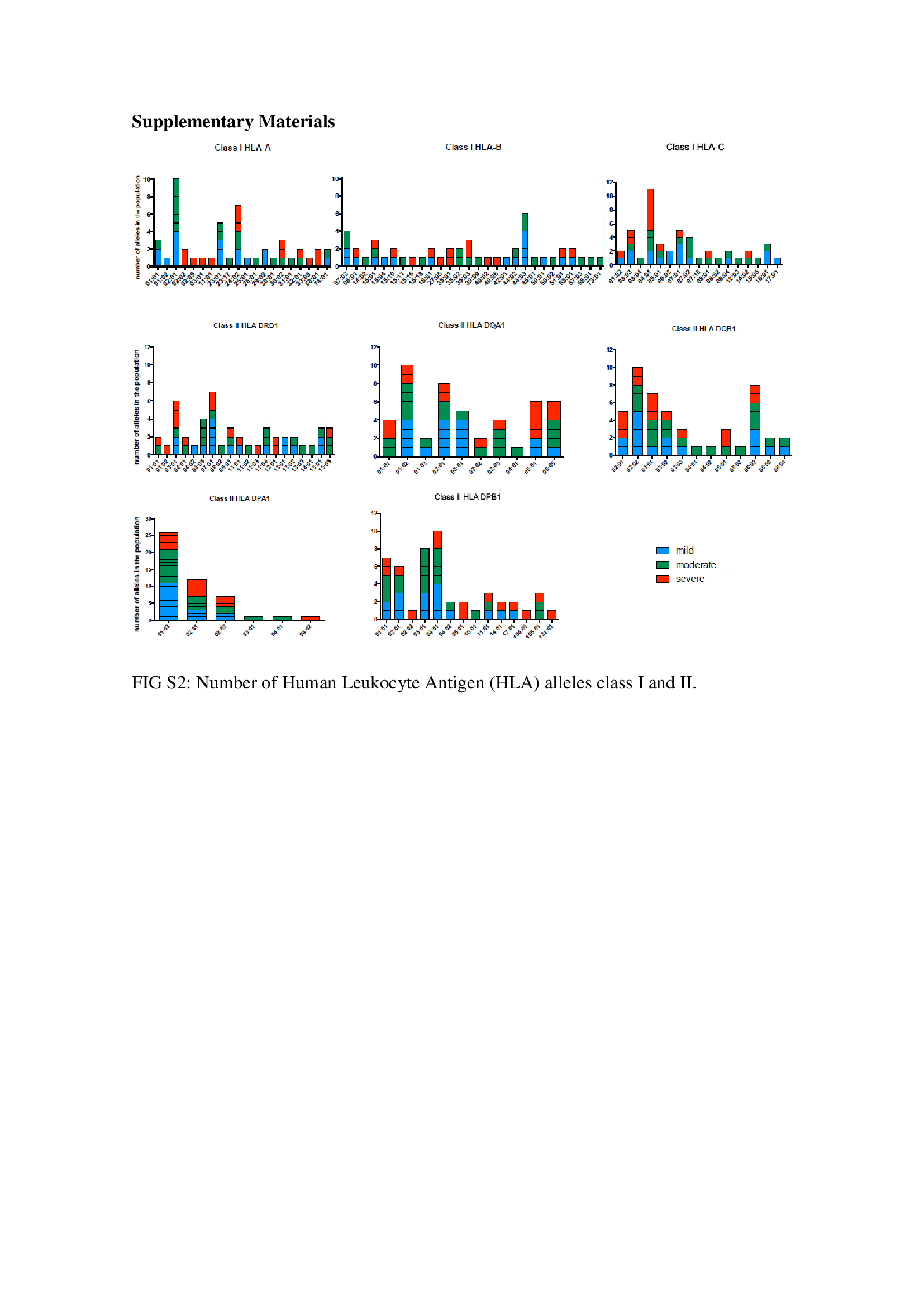

Supplement: Supplementary file 3 — Supplementary Material 3 [file 12879_2024_9490_MOESM3_ESM.jpg]
